# Enhancing Adherence to Chronic Heart Failure Monitoring: A Student-Led Quality Improvement Project During Clinical Placement

**DOI:** 10.7759/cureus.99217

**Published:** 2025-12-14

**Authors:** Matthew Severyn, Sarina Sanghera, Mazin Elmubarak, Aaliyah Ajibola, Seshnag Siddavaram

**Affiliations:** 1 Medicine, Guy's Hospital, King's College Hospital and St. Thomas' Hospital (GKT) School of Medical Education, King's College London, London, GBR; 2 Medicine, Guy's Hospital, King's College Hospital and St. Thomas' Hospital (GKT) School of Medical Education, King’s College London, London, GBR; 3 Acute Internal Medicine, Dartford and Gravesham NHS Trust, Dartford, GBR

**Keywords:** acute exacerbation of heart failure, chronic heart failure, clinical placement, heart failure management programmes, patient monitoring, pdsa cycles, physician guideline adherence, student-led initiatives, student research, undergraduate medical student

## Abstract

Introduction: Heart failure (HF) poses a major clinical and economic burden within UK hospitals, with poor inpatient monitoring often undermining guideline-directed medical therapy. In the Acute Medical Unit (AMU) at Darent Valley Hospital, pre-intervention audits revealed suboptimal adherence to National Institute for Health and Care Excellence monitoring standards for chronic heart failure (CHF). The areas of monitoring include functional capacity, fluid status, cognitive status using the Glasgow Coma Scale, nutritional status, urea and electrolytes (U+Es) and the locally recommended daily 12-lead ECG within the first 24 hours of admission. CHF monitoring adherence was assessed by scoring completion of these six predefined parameters. Completion of fluid status monitoring and nutritional status was particularly poor. This five-month quality improvement project (QIP), led by medical students, aimed to improve adherence to inpatient monitoring requirements for patients presenting with acute CHF decompensation (acute-on-chronic HF) within the first 24 hours of admission by implementing a CHF care bundle.

Methods: Using the Plan-Do-Study-Act (PDSA) model, we conducted two iterative intervention cycles. Baseline data were collected from 19 patients, followed by 13 and 19 patients in subsequent cycles. The intervention involved distributing a simplified care bundle flowchart to staff working in the AMU and providing orientation. Quantitative adherence data were collected from electronic health records and bedside documentation.

Results: Results showed a significant improvement in adherence to inpatient monitoring requirements, from a baseline median of 50.9%-86.8% after the second PDSA cycle. From baseline, the completion of nutritional status assessment (+52.6%), daily 12-lead ECG (+63.2%) and fluid status monitoring (+52.6%) saw the largest improvements. Run-chart analysis revealed that monitoring adherence stabilised by Cycle 2. Conclusion: This QIP demonstrates that cost-effective, low-resource, bundle-based interventions can enhance inpatient CHF monitoring, while showcasing the valuable role medical students can play in advancing sustainability initiatives within the NHS. Sustainability was supported by minimal reliance on additional infrastructure, enabling it to continue beyond the students’ placement period.

## Introduction

Heart failure (HF) is a clinical syndrome associated with impaired cardiac function and high morbidity, mortality and healthcare utilisation [1]. In the UK, HF affects around one million people and carries a five-year mortality rate of approximately 50% following diagnosis [2,3]. HF hospitalisations are rising in line with ageing demographics and increasing cardiovascular risk factors, placing mounting pressure on inpatient services [4,5]. While guideline-directed medical therapy significantly reduces mortality, its effectiveness depends on early and consistent inpatient clinical monitoring, particularly on the first day of acute exacerbation admission, of key factors such as activity level, fluid status and haemodynamic stability [6-10].

HF is therefore a common cause of hospital admission and a growing burden on NHS services due to increasing prevalence and population ageing [11]. At Darent Valley Hospital (DVH), a 478-bed district general hospital in Kent, the Acute Medical Unit (AMU) serves as the primary point of admission for patients requiring urgent medical review and short-term inpatient care. However, consistent adherence to National Institute for Health and Care Excellence (NICE)-recommended HF monitoring standards remains a challenge in busy clinical settings. Variability in monitoring practices is well documented and often worsened by high staff turnover and unclear ownership of daily tasks [12].

Baseline monitoring adherence (n = 19) on the AMU revealed inconsistent adherence to NICE chronic heart failure (CHF) monitoring standards within the first 24 hours upon admission [13]: functional capacity, fluid status, cognitive status, nutritional status, urea and electrolytes (U+Es) and locally recommended daily 12-lead ECG monitoring. This non-adherence was particularly evident in fluid status documentation (36.8%), nutritional status assessment completion (47.4%) and recommended 12-lead ECG monitoring (36.8%). From conducting Gemba walks in the AMU, it became clear that omissions were partly due to high patient volumes, frequent staff rotations and other reasons.

Care bundles have been widely adopted in quality improvement (QI) work to standardise care and improve outcomes. Lavallée et al. [14] and Roberti et al. [15] highlight that bundles comprising simple, evidence-based elements, when performed collectively, can enhance adherence to clinical best practices. In the context of HF, several studies have demonstrated measurable improvements following the implementation of a bundle. For example, Woodcock et al. [16] reported reductions in mortality and readmissions with an HF admission care bundle, whereas Edvinsson et al. [17] observed a 30-day readmission rate reduction from 30% to 5%.

Informed by this evidence, a quality improvement project (QIP) was initiated by medical students during their clinical placement at DVH, under the supervision of an internal medicine consultant. The project sought to address variation in HF monitoring by introducing a structured, low-cost HF care bundle aligned with NICE guidance [13]. This bundle aimed to improve monitoring standards for ‘Initial requirements’ and ‘Monitoring requirements’ for patients presenting with acute-on-chronic HF on the AMU by simplifying existing guidelines into a digital and physical bedside tool. We aimed to promote documentation practices and demonstrate that meaningful improvements can be achieved, even in resource-limited settings, by medical students at their placement site.

Our Specific, Measurable, Achievable, Relevant, and Time-bound aim, informed by established benchmarks in HF QI literature [9], was to increase adherence to recommended ‘Monitoring requirements’ in the first 24 hours of admission by 25% (from a baseline of 50.9%) by the end of our clinical placement in March 2025. An exploratory secondary aim was to improve adherence to the ‘Initial Clinical Assessment’ requirements by 10% (from 83.3%) over the same period.

## Materials and methods

**Figure 1:**
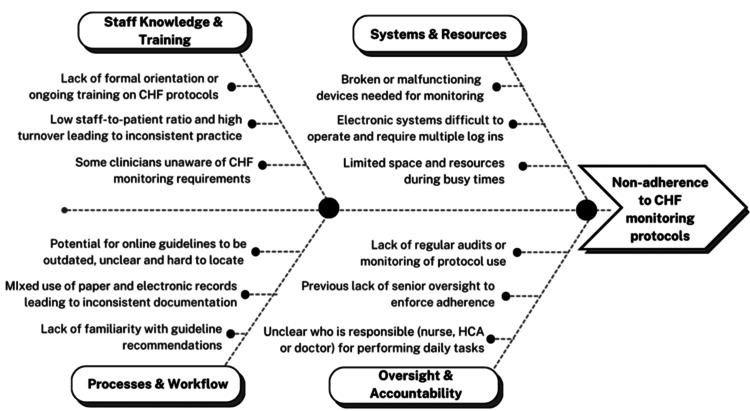
Fishbone diagram of contextual issues around CHF monitoring protocols CHF: chronic heart failure; HCA: healthcare assistant Image credit: This is an original image created by the authors Sarina Sanghera and Matthew Severyn

**Figure 2:**
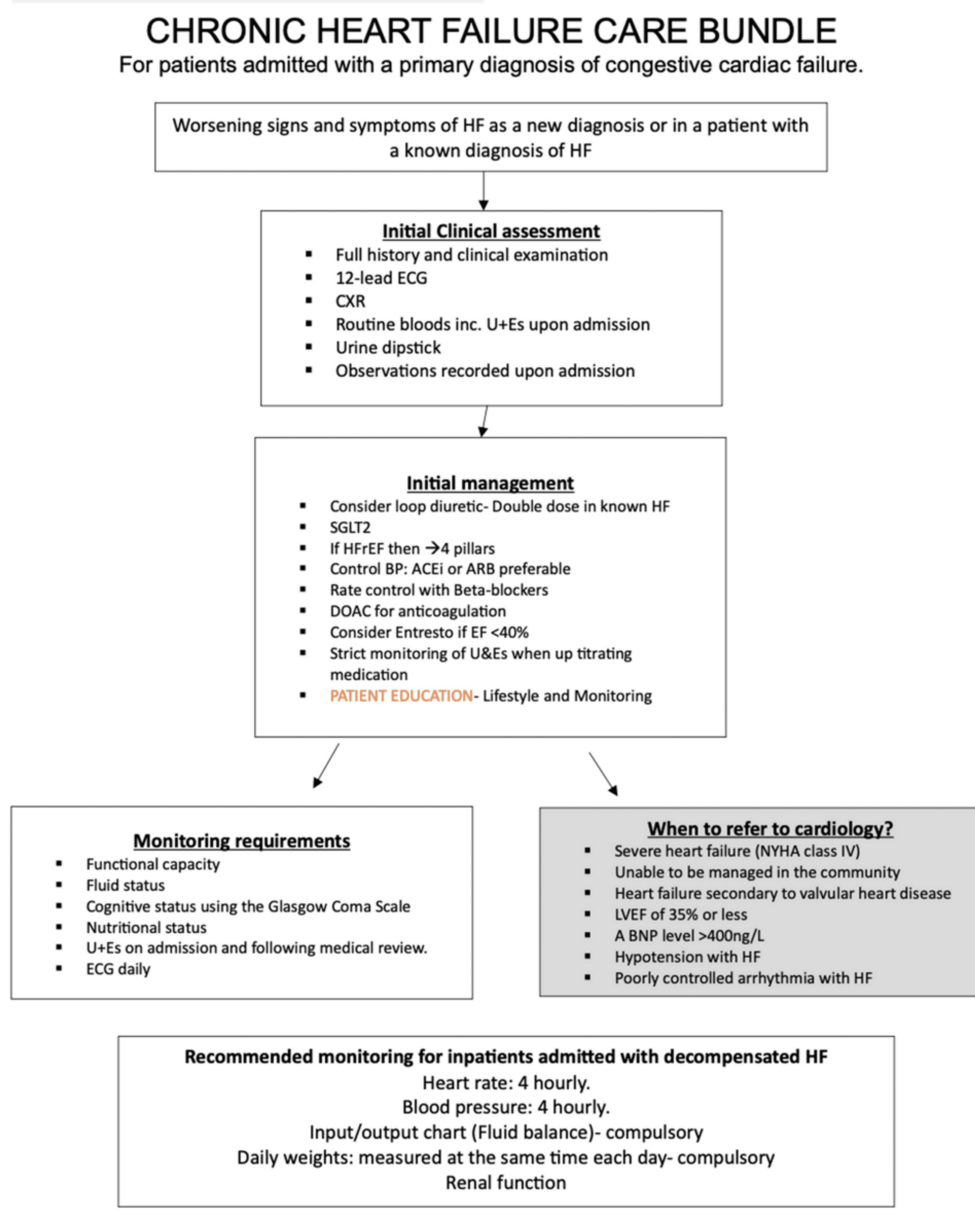
CHF care bundle HF: heart failure; CXR: chest X-ray; SGLT2: sodium-glucose cotransporter 2; BP: blood pressure; ACEi: angiotensin-converting enzyme inhibitor; ARB: angiotensin receptor blocker; DOAC: direct oral anticoagulant; EF: ejection fraction; NYHA: New York Heart Association; LVEF: left ventricular ejection fraction; BNP: brain natriuretic peptide; CHF: chronic heart failure Image credit: This is an original image created by the author Seshnag Siddavaram

Findings from Gemba walks were synthesised into a fishbone diagram and used to inform the design of the care bundle (Figure 1). Paper copies of the bundle were then distributed on the AMU, and digital versions of our care bundle were made available (see strategy) (Figure 2). Two process measures, one primary (A) and one exploratory (B), from our bundle were used to evaluate our intervention: (A) percentage adherence to six ‘CHF Monitoring Requirements’ (within the 24 hours of admission) and (B) percentage adherence to six ‘Initial Clinical Assessment’ (completed immediately on admission). These domains in the bundle were created based on NICE CHF guidelines [13], with these standards extrapolated to the acute-on-chronic HF setting. An additional locally recommended metric, daily 12-lead ECG monitoring, was included based on input from our supervising consultant. Continuous telemetry (three-lead) is standard on the ward, while daily 12-lead ECGs were introduced to enhance the early detection of evolving cardiac abnormalities [18]. Nutritional status was assessed using the Malnutrition Universal Screening Tool [19], a widely used tool for identifying adults at risk of malnutrition [20]. Cognitive status was assessed using the Glasgow Coma Scale (GCS) [21].

Participants and inclusion criteria

Eligible cases included adult (≥18 years) admissions to the AMU with a documented diagnosis of CHF presenting with acute decompensation (i.e., acute-on-chronic HF) and being present in the AMU during the designated daily two-week data collection periods. Each patient was monitored to determine whether the six ‘Monitoring Requirements’ were completed within the first 24 hours of admission, alongside their ‘Initial requirements’, reflecting the most acute period of stabilisation. No patient presented more than once during the monitoring period.

Exclusion criteria included 1) patients under 18 years old, 2) presentations in which HF was clearly secondary or incidental to another acute illness (e.g., pneumonia, chronic obstructive pulmonary disease exacerbation without HF decompensation) and 3) immediate transfers from the AMU to intensive or coronary care before completion of the initial 24-hour monitoring period.

Data handling and statistical analysis

For each eligible admission, completion of all 12 care-bundle elements (six Initial Clinical Assessment items and six Monitoring Requirements items) was recorded as present or absent in a spreadsheet using data extracted from the electronic health record and bedside documentation. Two adherence scores (0%-100%) were calculated per patient by dividing the number of completed items within each domain by the total number of items in that domain. Adherence data were analysed across three phases: baseline (September 2024), post-Plan-Do-Study-Act (PDSA) Cycle 1 (November-December 2024) and post-PDSA Cycle 2 (January-February 2025).

Given the modest sample size and student-run nature of this QIP, analyses were descriptive, and no formal hypothesis testing or power calculations were undertaken. Each phase included 13-19 patients. Adherence scores were plotted in chronological order by patient admission date, and temporal patterns were evaluated using the run-chart methodology. For each phase, domain-level adherence was summarised using means and medians, and results were presented as proportions with corresponding percentage-point changes between phases. Run charts were used to evaluate changes in ‘Monitoring Requirements’ adherence; individual patient scores were plotted sequentially, and median shifts were interpreted using established run-chart rules to identify non-random variation. Key process variables, including bundle launch dates, staff induction periods and weekday vs. weekend admissions, were tracked alongside trends to support interpretation of variation. All analyses and figure generation were conducted in RStudio version 2024.04.7 (R Foundation for Statistical Computing, Vienna, Austria) [22] with all visualisations produced using the ggplot2 package [23].

Data were collected daily each afternoon by four medical students working in alternating two-week rotations to ensure continuous project coverage. The project involved analysis of routinely collected, non-identifiable clinical data and met criteria for a service evaluation. In accordance with UK Health Research Authority guidance, formal Institutional Review Board or Research Ethics Committee approval was therefore not required [24].

Strategy

The intervention, a digitally available and printed CHF Care bundle, was developed by a team of four medical students during their clinical placement. The rationale for choosing a care bundle format was based on substantial evidence that bundle-based interventions improve clinical consistency, reduce errors of omission and support decision-making [14,16].

Given the high turnover of resident doctors and the busy environment of the AMU, we deliberately designed the intervention to be low-cost, low-tech and visually intuitive, requiring no additional infrastructure. To ensure the bundle's relevance and feasibility, we engaged stakeholders early through presentations at Clinical Governance and Grand Rounds.

The assumption underpinning the intervention was that cognitive overload and unclear accountability contributed to variation in practice at baseline [25]. A single-page, standardised checklist was expected to reduce this variation by serving as a mental prompt and aligning staff expectations.

Anticipated barriers included staff fatigue, competing clinical priorities and limited awareness among staff. To mitigate these, we included the bundle in staff handovers, inductions and governance discussions, and provided both paper and digital versions to maximise accessibility.

During the baseline phase in September 2024, we collected data on 19 patients to quantify current performance. Median adherence was 50.9% for ‘Monitoring Requirements’ and 83.3% for ‘Initial Clinical Assessment’. These findings confirmed significant variation in practice and the need for intervention. Our intervention was introduced during Grand Rounds and Clinical Governance presentations shortly after our initial cycle, with additional paper copies distributed on the AMU and digital versions of our care bundle shared via trust-wide email.

In PDSA Cycle 1 (November-December 2024), despite engagement, adherence to ‘Monitoring Requirements’ improved to only 66.7%, indicating that passive dissemination was insufficient to sustain practice change. We repeated governance presentations, conducted additional briefings during ward rounds and repeated email-wide correspondence. Following our intervention, in PDSA Cycle 2 (January-February 2025), monitored adherence increased further to 86.8%, indicating a positive shift in practice.

## Results

A total of 51 patients met the inclusion criteria across the three phases of data collection: 19 during baseline (September 2024), 13 after PDSA Cycle 1 (November-December 2024) and 19 after PDSA Cycle 2 (January-February 2025). The mean adherence to ‘Monitoring Requirements’ rose from 50.9% at baseline to 73.1% after PDSA Cycle 1 and ultimately to 86.8% after PDSA Cycle 2. ‘Initial Clinical Assessment’ adherence, by contrast, remained relatively static: 84.2% at baseline, 78.2% in Cycle 1, and 81.6% in Cycle 2 (Table 1).

**Table 1:** Detailed breakdown of adherence for each requirement at baseline and two PDSA cycles All percentages are rounded to one decimal place. Changes are presented as percentage point differences PDSA: Plan-Do-Study-Act

Initial assessment requirements	Baseline (n = 19)	PDSA cycle 1 (n = 13)	Δ from baseline (cycle 1)	PDSA cycle 2 (n = 19)	Δ from PDSA cycle 1	Δ from baseline (overall)
Full history and clinical examination	19/19 (100%)	13/13 (100%)	0%	19/19 (100%)	0%	0%
12-lead ECG	19/19 (100%)	13/13 (100%)	0%	19/19 (100%)	0%	0%
Chest X-ray	15/19 (78.9%)	9/13 (69.2%)	-8.6%	11/19 (57.9%)	-11.3%	-21.0%
Routine bloods inc. U+Es upon admission	19/19 (100%)	13/13 (100%)	0%	19/19 (100%)	0%	0%
Urine dipstick	5/19 (26.3%)	3/13 (23.1%)	-4.7%	6/19 (31.6%)	+8.5%	+5.3%
Observations recorded upon admission	19/19 (100%)	10/13 (76.9%)	-23.1%	19/19 (100%)	+23.1%	0%
Mean	84.2%	78.2%	-6%	81.6%	+3.4%	-2.6%
Monitoring requirements						
Functional capacity	8/19 (42.1%)	9/13 (69.2%)	+24.8%	10/19 (52.6%)	-16.6%	+10.5%
Fluid status	7/19 (36.8%)	13/13 (100%)	+61.1%	16/19 (84.2%)	-15.8%	+47.4%
Cognitive status using the Glasgow Coma Scale	12/19 (63.2%)	9/13 (69.2%)	+2.5%	17/19 (89.5%)	+20.3%	+26.3%
Nutritional status	9/19 (47.4%)	6/13 (46.2%)	-3.8%	19/19 (100%)	+53.8%	+52.6%
U+Es on admission and following medical review	15/19 (78.9%)	11/13 (84.6%)	+6.8%	18/19 (94.7%)	+10.1%	+15.8%
ECG daily	7/19 (36.8%)	9/13 (69.2%)	+30.3%	19/19 (100%)	+30.8%	+63.2%
Mean	50.9%	73.1%	+22.2%	86.8%	+13.7%	+35.9%

Initial clinical assessment domain

In our exploratory analysis (Table 1), adherence to the ‘Initial Clinical Assessment’ domain remained consistently high for full history, 12-lead ECG and admission blood tests, each achieving 100% completion across all cycles. Chest X-ray (CXR) adherence declined from 78.9% at baseline to 57.9% in Cycle 2, likely due to radiology backlogs during this period. Urine dipstick testing showed a modest improvement, increasing from 26.3% to 31.6%. Despite variability in individual components, the overall median adherence to ‘Initial Clinical Assessment’ remained stable at 83.3% across all PDSA cycles, and no meaningful improvement was observed in this domain.

Monitoring requirements domain

Significant improvements were observed in the ‘Monitoring Requirements’ domain (Table 1), with the mean number of completed components per patient increasing from 3.1 at baseline to 5.2 by PDSA Cycle 2. Fluid status monitoring rose sharply from 36.8% (7/19) at baseline to 100% (13/13) in Cycle 1, before settling at 84.2% (16/19) in Cycle 2. Completion of daily 12-lead ECGs improved from 36.8% at baseline to 100% by Cycle 2. Nutritional status assessment increased from 47.4% (9/19) to 100% (19/19), while functional capacity assessment showed a modest rise from 42.1% at baseline to 52.6% in Cycle 2.

**Figure 3:**
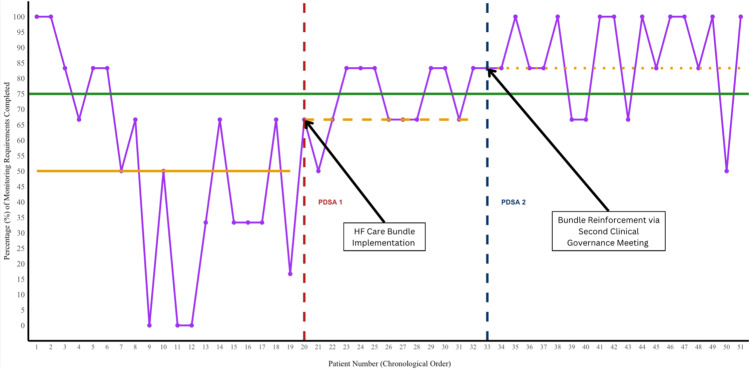
Run chart for quality improvement in “Monitoring Requirement” domain completion adherence (%) by patient (patient data collected chronologically from September 2024 to February 2025) The “Monitoring Requirements” domain includes functional capacity, fluid status, cognitive status, nutritional status, and U+Es on admission, followed by medical review and ECG daily. Orange: pre-intervention median (50.0%): PDSA1 median (66.7%) and PDSA2 median (83.3%); green: goal (75%) PDSA: Plan-Do-Study-Act

Run-chart analysis (Figure 3) revealed a mixed pattern for ‘Monitoring Requirements’. At baseline, six runs were observed, at the lower limit of the expected range (6-14), with a median adherence of 50.0%. During PDSA Cycle 1, the number of runs dropped to two, below the expected range (4-10), accompanied by an improved median of 66.7%, indicating special-cause variation likely driven by the intervention. In PDSA Cycle 2, adherence improved further, with seven runs (within the expected range) and a new median of 83.3%. Interestingly, we explored whether the day of admission influenced adherence, but no consistent pattern was observed. This suggests that weekday clinical pressures, such as post-weekend workload, did not have a clear or measurable impact on bundle adherence in our sample.

## Discussion

This project offers several lessons on how low-cost, student-led QI initiatives can drive measurable improvements in clinical practice. The first lesson is that simple interventions can change clinician behaviour, as literature shows that simple visual materials reduce cognitive load and improve workflow integration [26], all without requiring financial investment or significant digital infrastructure. Our team achieved a 35.9% mean improvement in adherence to CHF ‘Monitoring Requirements’, from 50.9% to 86.8%. Key gains from baseline were seen in areas such as fluid status documentation (+52.6%), daily 12-lead ECG completion (+63.2%) and nutritional status assessment (+52.6%). Importantly, these improvements were sustained into the second cycle, suggesting internalisation of the intervention. Second, the finding that some aspects of care were already well embedded is instructive. Adherence to the ‘Initial Clinical Assessment’ bundle remained high throughout, reflecting strong baseline familiarity among staff with core assessment practices. This aligns with the literature, which shows that established workflows are less amenable to significant incremental gains [27]. However, specific parameters, such as CXR completion, declined (-21.0% from baseline), likely due to radiology backlogs, illustrating how system-level pressures outside the immediate remit of the clinical team can limit QI impact and represent a methodological constraint of unit-level projects. Interdepartmental collaboration and system redesign may therefore be required to maximise impact [28].

We also learnt that our data did not reveal a consistent pattern linking adherence to the day of admission. This suggests that the CHF Care Bundle remained accessible and easy to use even during times of increased clinical workload, adding to the importance of designing clinical tools that remain effective and user-friendly even during periods of fluctuating clinical demand [29].

Another important lesson was the demonstration of how medical students, under structured supervision, can lead meaningful service improvement [30]. For example, a UK study found that 50% of student-led projects achieved significant improvements in primary outcomes [31]. By designing and delivering a QIP while attending our clinical placement, we were able to improve care while also gaining valuable experience in leadership, systems thinking and the principles of continuous QI. This project highlights the potential of integrating QI work into undergraduate medical education, benefiting not only students but also making real-time contributions to service delivery in the NHS [31].

A key strength of this QIP was the alignment of our intervention with the sustainable QI framework [32]. The intervention required no digital integration, minimising administrative burden and upfront infrastructure costs; hence, it was low-cost, low-impact on the environment, and promoted social sustainability by reducing documentation ambiguity and cognitive load for staff. Simplicity allowed easy integration into existing workflows, a feature cited as a facilitator of successful change [33]. To support long-term adoption, future iterations could involve more digital integration into the electronic health records and conducting repeat audits.

Limitations

Several limitations must be acknowledged. The project was conducted in a single NHS ward over a limited five-month period, which restricts generalisability. While run charts suggested sustained change in ‘Monitoring Requirements’, longer follow-up would be necessary to confirm true sustainability.

The relatively small sample size in each phase (13-19 patients per cycle) limits the statistical power of our findings. The Hawthorne effect [34] may have influenced adherence, particularly during the baseline phase, when staff were informed about impending data collection. We believe this is why adherence to monitoring protocols was initially high at baseline when we started, but declined within three days, suggesting short-term behavioural change in response to observation. Additionally, while informal feedback from staff was positive, the absence of formal qualitative interviews or surveys limited our ability to evaluate staff perspectives on the intervention. Cognitive status was assessed using the GCS [21], which, although not ideal for detecting subtle cognitive impairment [35] compared with tools such as the Montreal Cognitive Assessment [36,37], was used as a pragmatic alternative due to its routine availability in a fast-paced acute medical ward. This was important, as cognitive impairment in HF is associated with reduced quality of life and higher mortality [38]. We also acknowledge that 12-lead ECGs may not be feasible in all trusts; therefore, this metric should be considered a local recommendation and adapted according to available resources and clinical context.

Directions for future work

Our findings align with previous QIPs demonstrating improved HF care through structured care bundles [16,17] and contribute to the literature on how bundles can enhance both process and outcome measures when implemented reliably [15,16]. Future research should continue to focus on how simple bundles can drive change without additional resources and highlight their importance in resource-constrained NHS environments, where staff shortages and time pressures often limit the implementation of significant changes. Additionally, the project did not include patient-centred outcomes such as length of stay, readmission rates, mortality or patient experience, largely due to the time-limited, student-led nature of the work. Incorporating these measures in future work would offer clearer insight into the downstream clinical benefits of improved monitoring adherence. Nonetheless, as the bundle was derived directly from evidence-based recommendations, which are themselves associated with reductions in morbidity and mortality, it is reasonable to expect that improved adherence would translate into better patient outcomes. Finally, if repeated, the project would benefit from formal qualitative evaluation, longer term follow-up to assess sustainability and the inclusion of cross-departmental feedback loops (e.g., radiology) to address logistical challenges. Greater digital integration of the bundle into the electronic health record, along with the appointment of local champions to monitor adherence, could further strengthen adoption.

## Conclusions

This student-led QIP shows that a simple, low-cost CHF care bundle may improve CHF monitoring adherence in an acute medical setting. By integrating the intervention into routine workflows, adherence to key inpatient monitoring requirements increased substantially over a five-month period. Importantly, this project highlights how medical students can lead meaningful improvements during clinical placements while developing the ability to contribute to a more sustainable NHS.

## References

[1] Bozkurt B, Coats AJ, Tsutsui H (2021). Universal definition and classification of heart failure: a report of the Heart Failure Society of America, Heart Failure Association of the European Society of Cardiology, Japanese Heart Failure Society and Writing Committee of the Universal Definition of Heart Failure: endorsed by the Canadian Heart Failure Society, Heart Failure Association of India, Cardiac Society of Australia and New Zealand, and Chinese Heart Failure Association. Eur J Heart Fail.

[2] Conrad N, Judge A, Tran J (2018). Temporal trends and patterns in heart failure incidence: a population-based study of 4 million individuals. Lancet.

[3] Yancy CW, Jessup M, Bozkurt B (2017). 2017 ACC/AHA/HFSA focused update of the 2013 ACCF/AHA guideline for the management of heart failure: a report of the American College of Cardiology/American Heart Association Task Force on Clinical Practice Guidelines and the Heart Failure Society of America. J Am Coll Cardiol.

[4] Mosterd A, Hoes AW (2007). Clinical epidemiology of heart failure. Heart.

[5] McDonagh TA, Metra M, Adamo M (2021). 2021 ESC guidelines for the diagnosis and treatment of acute and chronic heart failure. Eur Heart J.

[6] Yang SH, Mu PF, Wu HL, Curia M (2019). Fluid balance monitoring in congestive heart failure patients in hospital: a best practice implementation project. JBI Database System Rev Implement Rep.

[7] Heidenreich PA, Bozkurt B, Aguilar D (2022). 2022 AHA/ACC/HFSA guideline for the management of heart failure: a report of the American College of Cardiology/American Heart Association Joint Committee on Clinical Practice Guidelines. Circulation.

[8] Reynolds K, Butler MG, Kimes TM, Rosales AG, Chan W, Nichols GA (2015). Relation of acute heart failure hospital length of stay to subsequent readmission and all-cause mortality. Am J Cardiol.

[9] Bakhai S, Bhardwaj A, Phan H, Varghese S, Gudleski GD, Reynolds JL (2019). Optimisation of diagnosis and treatment of heart failure in a primary care setting. BMJ Open Qual.

[10] Ali D, Banerjee P (2017). Inpatient monitoring of decompensated heart failure: what is needed?. Curr Heart Fail Rep.

[11] Cowie MR (2017). The heart failure epidemic: a UK perspective. Echo Res Pract.

[12] Nilsen P, Seing I, Ericsson C, Birken SA, Schildmeijer K (2020). Characteristics of successful changes in health care organizations: an interview study with physicians, registered nurses and assistant nurses. BMC Health Serv Res.

[13] (2025). Heart failure - chronic: clinical knowledge summary. https://cks.nice.org.uk/topics/heart-failure-chronic/.

[14] Lavallée JF, Gray TA, Dumville J, Russell W, Cullum N (2017). The effects of care bundles on patient outcomes: a systematic review and meta-analysis. Implement Sci.

[15] Roberti J, Vita T, Piastrella J (2020). Care bundle to reduce readmission in patients with heart failure: a modified Delphi consensus panel in Argentina. BMJ Open.

[16] Woodcock T, Matthew D, Palladino R (2023). Effect of implementing a heart failure admission care bundle on hospital readmission and mortality rates: interrupted time series study. BMJ Qual Saf.

[17] Edvinsson ML, Stenberg A, Åström-Olsson K (2019). Improved outcome with standardized plan for clinical management of acute decompensated chronic heart failure. J Geriatr Cardiol.

[18] Sattar Y, Chhabra L (2025). Electrocardiogram.

[19] Marinos E (2003). Nutritional Screening of Adults: A Multidisciplinary Responsibility. Redditch: British Association for Parenteral and Enteral Nutrition.

[20] Stratton RJ, Hackston A, Longmore D (2004). Malnutrition in hospital outpatients and inpatients: prevalence, concurrent validity and ease of use of the 'Malnutrition Universal Screening Tool' ('MUST') for adults. Br J Nutr.

[21] Teasdale G, Jennett B (1974). Assessment of coma and impaired consciousness: a practical scale. Lancet.

[22] (2025). RStudio: integrated development environment for R. https://www.posit.co/.

[23] Wickham H (2025). ggplot2: elegant graphics for data analysis (software). https://ggplot2.tidyverse.org/.

[24] Health Research Authority (2025). What approvals and decisions do I need?. https://www.hra.nhs.uk/approvals-amendments/what-approvals-do-i-need.

[25] Dingley C, Daugherty K, Derieg MK (2008). Improving patient safety through provider communication strategy enhancements. Advances in Patient Safety: New Directions and Alternative Approaches.

[26] Baxter KA, Sachdeva N, Baker S (2025). The application of cognitive load theory to the design of health and behavior change programs: principles and recommendations. Health Educ Behav.

[27] Foy R, Skrypak M, Alderson S (2020). Revitalising audit and feedback to improve patient care. BMJ.

[28] He Z, An M, Chen D, Peng H, Tao H, Cheung KM (2025). Institution-based quality and safety improvement initiatives in spine surgery: a scoping review. JBJS Rev.

[29] Olakotan O, Samuriwo R, Ismaila H, Atiku S (2025). Usability challenges in electronic health records: impact on documentation burden and clinical workflow: a scoping review. J Eval Clin Pract.

[30] Dolfini L, Williamson G, Oakeshott P (2020). Including medical students in quality improvement projects in primary care. Future Healthc J.

[31] Radenkovic D, Mackenzie R, Bracke S, Mundy A, Craig D, Gill D, Levi M (2019). Involving medical students in service improvement: evaluation of a student-led, extracurricular, multidisciplinary quality improvement initiative. Adv Med Educ Pract.

[32] (2025). The SusQI education project: putting theory into practice. https://sustainablehealthcare.org.uk/activity/the-susqi-education-project-putting-theory-into-practice/.

[33] Cavalcanti DR, Oliveira T, de Oliveira Santini F (2022). Drivers of digital transformation adoption: a weight and meta-analysis. Heliyon.

[34] Allen RL, Davis AS (2011). Hawthorne effect. Encyclopedia of Child Behavior and Development.

[35] Dong P, Cremer O (2011). Limitations of the use of the Glasgow Coma Scale in intensive care patients with non-neurological primary disease: a search for alternatives. Crit Care.

[36] McLaren J, Fradera A, Cullen B (2025). The reliability and validity of brief cognitive screening tools used in traumatic brain injury: a systematic review. Neuropsychol Rehabil.

[37] Nasreddine ZS, Phillips NA, Bédirian V (2005). The Montreal Cognitive Assessment, MoCA: a brief screening tool for mild cognitive impairment. J Am Geriatr Soc.

[38] Kewcharoen J, Prasitlumkum N, Kanitsoraphan C, Charoenpoonsiri N, Angsubhakorn N, Putthapiban P, Rattanawong P (2019). Cognitive impairment associated with increased mortality rate in patients with heart failure: a systematic review and meta-analysis. J Saudi Heart Assoc.

